# What the newspapers say about medication adherence: a content analysis

**DOI:** 10.1186/1471-2458-13-909

**Published:** 2013-10-02

**Authors:** Nicola A Goodfellow, Basima A Almomani, Ahmed F Hawwa, James C McElnay

**Affiliations:** 1 Clinical and Practice and Research Group, School of Pharmacy, Queen’s University Belfast, 97 Lisburn Road, Belfast BT9 7BL, Northern Ireland; 2 Department of Clinical Pharmacy, Jordan University of Science and Technology, Irbid 22110, Jordan

**Keywords:** Newspaper article, Newspapers, Patient compliance, Adherence

## Abstract

**Background:**

This study investigates the coverage of adherence to medicine by the UK and US newsprint media. Adherence to medicine is recognised as an important issue facing healthcare professionals and the newsprint media is a key source of health information, however, little is known about newspaper coverage of medication adherence.

**Methods:**

A search of the newspaper database Nexis®UK from 2004–2011 was performed. Content analysis of newspaper articles which referenced medication adherence from the twelve highest circulating UK and US daily newspapers and their Sunday equivalents was carried out. A second researcher coded a 15% sample of newspaper articles to establish the inter-rater reliability of coding.

**Results:**

Searches of newspaper coverage of medication adherence in the UK and US yielded 181 relevant articles for each country. There was a large increase in the number of scientific articles on medication adherence in PubMed® over the study period, however, this was not reflected in the frequency of newspaper articles published on medication adherence. UK newspaper articles were significantly more likely to report the benefits of adherence (p = 0.005), whereas US newspaper articles were significantly more likely to report adherence issues in the elderly population (p = 0.004) and adherence associated with diseases of the central nervous system (p = 0.046). The most commonly reported barriers to adherence were patient factors e.g. poor memory, beliefs and age, whereas, the most commonly reported facilitators to adherence were medication factors including simplified regimens, shorter treatment duration and combination tablets. HIV/AIDS was the single most frequently cited disease (reported in 20% of newspaper articles). Poor quality reporting of medication adherence was identified in 62% of newspaper articles.

**Conclusion:**

Adherence is not well covered in the newspaper media despite a significant presence in the medical literature. The mass media have the potential to help educate and shape the public’s knowledge regarding the importance of medication adherence; this potential is not being realised at present.

## Background

Adherence has gained recognition as one of the most important issues facing the healthcare community [1] and improving adherence to treatment is regarded as being the single most important health intervention likely to improve the health of the population when compared with advancing any individual treatment [2]. Poor adherence to treatment represents a significant problem and can be detrimental to patient health outcomes and the healthcare economy. Non-adherence can lead to a lack of improvement or worsening of disease state resulting in increased healthcare costs, for example, due to preventable hospitalisation’s, avoidable disease complications and unused medication [3]. Furthermore, medication adherence can be influenced by beliefs about medicines [4-7]. These beliefs are, in part, informed by the newsprint media [8].

Newspapers disseminate substantial amounts of information about medicines and health to the general public on a regular basis [8]. The content of newspaper reporting should be of significant interest to the medical community as it has been observed that, *“although the media may not have the power to determine what people think, they can and do determine what people think about”*[9].

The mass media has the ability to deliver messages to a large proportion of the population. There are a number of different sources providing health information to the public including healthcare professionals, Internet, television and radio, however, studies show that newspapers continue to be a key source in the dissemination of health information [10]. A recent survey reported that 35.2% of adults in Great Britain had read at least one daily newspaper within the previous day [11] and as such the mass media have previously been employed in communicating health promotion messages to the public with varying degrees of success e.g. improving health services utilisation [12], immediate improvement on the uptake of HIV testing [13] and inconsistent effects on smoking cessation [14]. Newspaper coverage has also been shown to impact reader’s attitudes towards public health interventions such as the smoking ban in The Netherlands [15]. Furthermore, a number of studies have investigated a range of health issues highlighted in newspapers, for example, how medicines are portrayed in the media [8], paediatric medication safety [16], medication errors [17], the uptake of the MMR vaccination [18] and the uptake of breast cancer screening [19].

There is no published research investigating newspaper reporting of treatment adherence, therefore, the aim of the present study was to investigate what has been communicated to the public in the UK and US about this important issue in terms of the frequency, content and context of the information provided.

## Methods

### Study design

**Figure 1:**
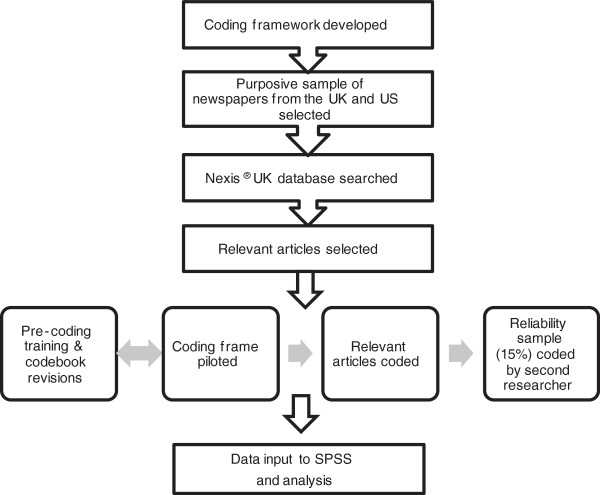
Overview of content analysis methodology.

We performed a content analysis of newspaper articles citing medication adherence published between 1st January 2004 and 31st December 2011. An overview of the methodological approach used in the present study is illustrated in Figure 1.

### Newspaper selection

We used the newspaper database Nexis®UK (2012) to retrieve archived newspaper articles which addressed medication adherence in a total of 12 UK newspapers (two Sunday papers and ten daily newspapers, together with their Sunday equivalents). We selected these newspapers since they had the highest average net circulation per issue at the time of commencement of data collection [20] (April 2010). They provided a broad spread of readership, from broadsheet to tabloid and from conservative to socialist in political outlook. The newspapers searched were; *The Sun, Daily Mail (Mail on Sunday), The Mirror (The Sunday Mirror), Daily Record (Sunday Mail), The Daily Telegraph (The Sunday Telegraph), The Times (The Sunday Times), The Express (The Sunday Express), Daily Star (Sunday Star), The Guardian (The Observer), Financial Times, The People* and *The News of the World.* Similarly, a purposive sample of the top twelve daily US newspapers ranked by total average paid circulation (six month average ending September 30th 2009) [21] were also searched *i.e. The New York Post, Chicago Tribune, The New York Times, USA Today, Los Angeles Times, The Houston Chronicle, The Washington Post, Wall Street Journal, Daily News (New York), The Philadelphia Inquirer, San Jose Mercury News* and the *Detroit Free Press* for data retrieval. All except the Wall Street Journal and USA Today were published on Sundays. Limitations of the Nexis®UK search engine included; the ability to search only the previous 6 months of articles in the LA Times and only abstract versions of Wall Street Journal articles were available to access.

### Search strategy and eligibility criteria

After empiric testing of various search terms, the search terms used were [“adherence OR compliance OR concordance” AND “drug OR medic! OR treatment”]. The ! symbol represented a wild card search and therefore medic! included medication(s), medical and medicine(s). Efforts to increase the specificity of search terms used resulted in non-selection of a proportion of relevant articles on medication adherence, therefore broad search terms were employed to minimise the loss of relevant articles.

We set criteria for the inclusion and exclusion of retrieved articles for detailed analysis. Articles were initially included if they contained any reference to patient adherence to conventional medicines or medical treatments, including articles in any format (for example, news article, editorial, letters to the editor). We excluded retrieved articles if their focus was on adherence to complementary or alternative therapies (such as herbal or homeopathic remedies), adherence to medical procedures or immunisation strategies, adherence to guidelines rather than medicines (for example, healthcare regulatory compliance) or drug testing, or if the article included reference to adherence only as part of an announcement (for example, advertising a workshop or conference on adherence). We addressed duplication of articles (for example, in the daily and Sunday editions of the same newspaper) by only including the article with the highest word count in the analysis.

NG selected the relevant articles and if there was indecision as to the inclusion of a newspaper article, two other researchers BA and JMcE were consulted and a consensus reached on whether the article met the inclusion requirements.

### Data extraction

We developed an *a priori* coding frame allowing data from each relevant article to be extracted and classified. The coding frame was adapted from previously published studies [22,23] and two researchers (NG and BA) piloted this using 10 articles. During this pilot the coding frame was adjusted slightly to enhance its specificity. Once finalised, the main researcher (NG) coded all the relevant articles. Newspaper article bibliographic details such as the newspaper name, article title, date, and author were recorded in the coding frame. Additionally, we recorded pertinent information contained within each article, such as whether adherence was linked to a medicine or disease, whether the article stated benefits of adherence and/or the harms of non-adherence, barriers or facilitators to adherence and the main source of adherence information. We also recorded further variables including the article slant which was classified as positive if adherence to medicine was viewed as beneficial, neutral if only factual information about adherence was presented or mixed if the article referred to adherence being both beneficial and harmful. The type of non-adherence was also described, for example intentional or unintentional. The quality of information presented on adherence was also assessed and assigned a subjective rating between 1 (poor) to 10 (excellent). A poor quality article, for example, may have contained only a short statement about adherence, whereas an article categorised as high quality would have included definitive information about adherence, for example, the inclusion of a definition of adherence, have a scientific article as an information source or include information about barriers or facilitators of adherence. If the newspaper article mentioned a scientific journal article as a source of information, we recorded details from the scientific article, for example, the journal reference, study design and disclosure of conflicts of interest.

To allow assessment of coding consistency a second researcher (BA) coded a 15% random sample of relevant articles from the UK and US. We used Cohen’s *kappa* statistic to determine the level of agreement between coders for questions with mutually exclusive answers.

Following data extraction, data were entered into IBM SPSS (version 19, SPSS Inc, USA) for analysis. This allowed the identification of trends in newspaper content and the comparison of different variables over time and between countries. Differences in the reporting of categorical variables in articles published in the UK and the US were assessed using the χ^2^ or the Fisher’s Exact test, as appropriate. Continuous variables were assessed using the Mann–Whitney U test. The level of statistical significance was set at 0.05. To compare the frequency of scientific articles published with reports published in newspapers we carried out an advanced search on PubMed® for each year over the period studied using the MeSH term ‘patient compliance’ which includes various terms such as patient adherence, non-adherence and medication adherence.

## Results

**Figure 2:**
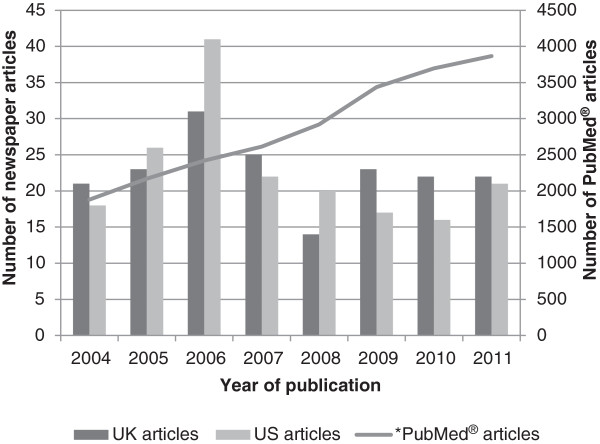
**Frequency of newspaper and scientific articles published about medication adherence annually.** * Scientific articles indexed using (Patient compliance [MeSH Term]) in the PubMed® database.

The initial searches yielded a total of 3,966 UK and 6,017 US newspaper articles over the period evaluated. From these, 181 articles from the UK and, coincidentally, 181 from the US met the study criteria. The number of relevant newspaper articles pertaining to adherence published annually remained relatively constant over the 8 year investigation period except for an increase, notably in American newspapers, in 2006. The number of scientific articles archived by PubMed® which were indexed using the patient compliance MeSH term increased each year over the same period as shown in Figure 2 to a maximum of nearly 4,000 articles in 2011. The inter-rater reliability *kappa* values for the coders were found to be within the acceptable range 0.54-0.96 [16,19,23,24].

Adherence was the main focus in only 16% of the articles included in the detailed analyses. In the cases where adherence was the main focus, health outcomes and research were the principal themes of the articles. The proportion of each article (percentage word count) that dealt specifically with the topic of adherence was low, with a median of 11% (range 0.25-100%).

In general the terms *“adherence”*, *“compliance”* and *“concordance”* were used inter-changeably, however, *“compliance”* was used most commonly with 349 mentions compared to adherence (181) and concordance (5). Despite being used interchangeably these words have distinct meanings [1,3]. Attempts within articles to define the terminology used occurred only 9 times for compliance, 7 times for adherence and 3 times for concordance.

The benefits of adhering to medication as prescribed were stated in 34% of the articles overall. Improved health outcome was the benefit stated most frequently (72% of articles), for example, *“Early diagnosis together with prevention and compliance with treatment are the essentials for the control of tuberculosis”* (Stuttaford T. New test may curb TB surge. The Times. 26 Aug 2004; Features: Times2, p14). Economic considerations were identified as a secondary key theme in relation to adherence which included factors such as paying for prescriptions *“A new study published on Monday shows patients are more likely to take their medicine when they do not have to help pay for the prescriptions”* (Abelson R. Study finds co-payments discourage drug treatments. The New York Times. 14 Nov 2011; Health) and general medication wastage *“…examples of waste in Scotland's health service include an estimated £18 m on unused prescription medicines. In Tayside alone prescription medicines worth £1.2 m are being issued to patients but never used”* (Allardyce J. Health service ‘squandering £1bn a year’. The Sunday Times. 25 Mar 2007; Home News, p5). The frequency of reporting benefits of adherence was significantly higher in UK newspaper articles (41%) compared to those published in the US (27%; chi-square analysis; p = 0.005).

One third of articles reported harms associated with non-adherence. In those cases where harmful consequences of non-adherence were reported, worse health outcome was stated in the majority of articles (62%), followed by worse economic outcome (30%). There was no significant difference between UK and US in the frequency of reporting of harms of non-adherence (Chi-square analysis; p > 0.05). The following is an illustrative example of these harms of non-adherence: *“*[Poor adherence can lead to] *troublesome symptoms, time off work and treatment in hospital …Poor compliance creates a significant economic burden too, one that the NHS can ill afford. Some inhalers are hugely expensive”* (Porter M. The penalty you pay for not tackling asthma properly. The Times. 30 Nov 2009; Features; T2, p9).

There was no significant difference between the UK and US in the reporting frequency of barriers or facilitators to adherence (Chi-square analysis; p > 0.05). With regards to factors reported to act as barriers to medication adherence, the most frequently cited were patient related (55%), such as poor memory, beliefs (cultural, religious, disease and medication beliefs) and age (e.g. adolescents being less adherent). Facilitators of adherence were stated in 66% of the articles; medication factors such as simplified regimens, shorter treatment duration, new medications and combination tablets were the most frequently reported facilitators.

The patient age group to which adherence information in the newspaper articles related was specified in only 28% of the articles. The elderly population were significantly more likely to be cited in US articles (14%) when compared with articles from UK newspapers (9%; Chi-square analysis; p = 0.004).

A specific medicine was linked to commentary on adherence in 47%, while a specific disease was specified in 77% of the articles. Medicines and diseases were classified according to the relevant chapter in the British National Formulary [25] during the analysis. The most frequently stated disease and treatment type related to the cardiovascular and infection categories. HIV/AIDS was the single most frequently cited disease and was reported in 73 articles (20%). There was a significant difference between UK and US newspaper reporting of adherence issues in diseases affecting the central nervous system (Chi-square analysis; p = 0.046), with a higher frequency reported by US newspapers (20% vs. 12%). Adherence reporting among other disease groups did not differ significantly between UK and US newspapers.

Adherence to therapy was overwhelmingly described as a positive health behaviour, with 84% of articles classified as having a positive slant on adherence. Nevertheless, 14% of the articles were written using a neutral slant when adherence was reported only in a factual manner and 2% reported mixed positive and negative viewpoints. In newspaper articles which discussed inappropriate prescribing, adherence was viewed as a negative health behaviour (i.e. adherence to inappropriate medication was potentially harmful).

Intentional non-adherence was the most frequently reported type of non-adherence in the present study with 17% of newspaper articles implying intentional non-adherence. By way of comparison, 14% of articles referred to unintentional non-adherence alone. Both intentional and unintentional non-adherence was implied in 15% of newspaper articles.

The quality of the information presented about medication adherence was analysed by the coders on a scale of 1–10, low to high quality respectively. On average, the quality was low; with articles scoring a mean of 3.4 out of 10. The quality score was not significantly different between countries (Mann–Whitney U analysis; p > 0.05). Overall, 62% of articles were classified as providing poor quality information on adherence (scored 1–3), 30% presented average/good quality reporting on adherence (scored 4–7) and only 8% were graded as reporting excellent quality information on adherence (scored 8–10).

## Discussion

Non-adherence to prescribed medication is a common problem witnessed by many healthcare professionals treating many different diseases. The data presented here is the first study to explore medicine adherence reporting by newspapers which continue to be an important source of health information for the public.

### Frequency and quality of reporting

Information about medicine adherence in the media was not frequently reported. The low coverage of treatment adherence was surprising due to the amount of dedicated research within this scientific area [26] (Figure 2). Furthermore, only 14% of articles specifically cited a scientific study as the main source of information. This study therefore demonstrates that adherence is not well covered in the newspaper media despite a significant presence in the medical literature.

The frequency of relevant newspaper articles increased in 2006 in the UK and US. This increase was more apparent in the US and was mostly due to the Food and Drug Administration approval of two fixed dose combination antiretroviral drugs for HIV/AIDS: a generic lamivudine-zidovudine-nevirapine product in June and Atripla® (efavirenz-emtricitabine-tenofovir disoproxil) in July 2006. These were both accompanied by FDA press releases and Atripla® was also press released by the manufacturer.

The quality of reporting about adherence in newspapers in the present study was generally classified as poor. The quality of newspaper reporting about health issues has been deemed poor, with the populist tabloid press singled out as the worst offender [27-29]. A study by Schwartz and colleagues [30] has shown that high quality newspaper articles were associated with a high quality press releases from a scientific journal. Despite the large number of scientific journal articles published on medication adherence the low quality of newspaper reporting on medication adherence suggests that press releases on adherence studies are not being brought forward by researchers/journals or that newspaper editors are choosing not to report the findings of adherence studies, presumably due to a perceived lack of public interest. In order to maximise uptake, we recommend that press releases should use lay language which could be directly incorporated into newspaper articles, without the need for too much editorial interpretation.

### Context of medication adherence reported by newspaper articles

Over the years many different terms have been used to describe the issue of deviating from prescribed treatments. The term *“compliance”* was used most frequently in newspaper articles despite it being reflective of a paternalistic model of care and the extensive use of the term “*adherence*” in current medical literature. This finding was surprising as adherence is the preferred term used in the medical and pharmaceutical literature [31].

There is some degree of controversy in the literature about whether newspapers are biased in their reporting of ‘bad’ news stories in comparison to ‘good’ news stories, for example, adverse drug reactions compared to the benefits of new and existing medicines. Good news stories regarding the use of medicines compared with bad news stories have, however, been shown to be more likely to be reported by the Dutch media [32] and this positive portrayal of medicines was supported by a more recent UK study [33]. Conversely, Bartlett *et al.*[34] found that bad news regarding medical research was more likely to be reported in British newspapers and Prosser [8] reported that new medicines were presented as low risk whereas, higher risks were reported for established medicines. In the present study the benefits of adherence (good news) and the harms of non-adherence (bad news) were reported equally overall, however, UK newspapers were significantly more likely to report the benefits of adherence when compared to newspaper articles published in the US.

Detrimental economic outcome was a commonly reported harm of non-adherence to therapy. A decline in health due to non-adherence can lead to the avoidable use of expensive resources such as hospitalisations, the use of costlier and/or unnecessary second line medication and wastage through unused medications [3]. The economic implications of non-adherence are particularly relevant in the current economic climate when, for example, the NHS has been tasked to make savings of between £15-20 billion between 2011 and 2015 [35]. The cost of hospital admissions alone resulting from non-adherence to medications in the UK has been estimated to be as high as £196 million in 2006–2007 [36].

The most frequently reported barrier to adherence stated within newspaper articles were patient related, encompassing factors such as patient held beliefs, poor manual dexterity, low motivation, poor knowledge and age. Barriers to treatment vary with each individual, treatment and disease. There is a growing body to support patient held beliefs about medicine as contributing to a significant amount of variance in adherence [4-7]. The most frequently cited facilitators for adherence related to medication factors which corresponded with treatment adherence facilitators proposed by the National Institute for Health and Clinical Excellence [3] and included providing more palatable formulations, less frequent dosing and fixed-dose combination products.

HIV/AIDS was the single most frequently reported disease; this perhaps reflects the severe consequences of non-adherence despite the relatively low prevalence of HIV/AIDS in the UK and US [37]. Adherence to antiretroviral therapy must exceed 95% to promote successful virologic outcome and slow disease progression [38,39]. This high level of adherence required is a stark contrast to the 50% estimated rate of adherence to therapy in most chronic diseases [2].

Examination of the extent of intentional and unintentional non-adherence in vulnerable populations has been highlighted as an area requiring further research [1]. Unintentional non-adherence has been shown to account for 55% of non-adherence in patients taking a new medication for a chronic condition [40]. In the present research, reported drivers of intentional non-adherence included factors such as beliefs (beliefs about medicines, disease, cultural beliefs or religious beliefs) and side effects. Identified drivers of unintentional non-adherence included, forgetfulness, access to medicine, illness-related confusion, poor health literacy and poor inhaler technique.

### Limitations

Newsprint media was the only media surveyed; other media sources which may be important to the public were not analysed such as radio or television, however, published data suggest that the coverage of news stories are highly correlated across all areas of the mass media [41]. Additionally, other sources of health information such as advice and brochures from healthcare professionals were not analysed. The Nexis®UK newspaper database is widely used in research literature, however, the database itself has limitations firstly, out of the newspapers included in the database, complete coverage of every article is not achieved due to, for example, copyright restrictions [16].

## Conclusions

Newspaper media does not adequately cover the important issue of medication adherence. The mass media has the potential to help educate and shape the public’s knowledge regarding medication adherence; this potential is not being realised at present. Authors of research articles on adherence should be pro-active in providing press releases on their work to help encourage coverage by newspapers of this important health issue.

## Competing interests

The authors declare that they have no competing interests.

## Authors’ contributions

NG was involved in the design, acquisition and analysis of data, and drafting the manuscript. BA was involved in design, data acquisition and drafting the manuscript. AH was involved in design and commenting on each draft of the manuscript. JMcE was involved in study conception, design, commenting on each draft of the manuscript. JMcE is the guarantor. All authors approved the final version of the manuscript.

## Pre-publication history

The pre-publication history for this paper can be accessed here:

http://www.biomedcentral.com/1471-2458/13/909/prepub
